# Intense zonal freshwater transport in the Eurasian Arctic during ice-covered season revealed by in situ measurements

**DOI:** 10.1038/s41598-023-43524-w

**Published:** 2023-10-02

**Authors:** Alexander Osadchiev, Roman Sedakov, Dmitry Frey, Alexandra Gordey, Vladimir Rogozhin, Zinaida Zabudkina, Eduard Spivak, Ekaterina Kuskova, Andrey Sazhin, Igor Semiletov

**Affiliations:** 1 Shirshov Institute of Oceanology, Russian Academy of Sciences, Moscow, Russia; 2 Moscow Institute of Physics and Technology, Dolgoprudny, Russia; 3 Marine Research Center at Lomonosov Moscow State University, Moscow, Russia; 4 Marine Hydrophysical Institute, Russian Academy of Sciences, Sevastopol, Russia; 5 Ilyichev Pacific Oceanological Institute, Far Eastern Branch of the Russian Academy of Sciences, Vladivostok, Russia; 6 Lomonosov Moscow State University, Moscow, Russia

**Keywords:** Physical oceanography, Physical oceanography

## Abstract

The Kara Sea receives ~ 1/3 of total freshwater discharge to the Arctic Ocean, mainly from the large Ob and Yenisei rivers. The Ob-Yenisei plume covers wide area in the central part of the Kara Sea during ice-free season (June–October) and accumulates ~ 1000 km^3^ of freshwater volume. In late autumn, the Kara Sea becomes covered by ice, which hinders in situ measurements at this area. As a result, the fate of the Ob-Yenisei plume below sea ice during winter and spring remains unclear. In this study, we report multiple in situ measurements performed in the Kara Sea shortly before and during ice-covered season. We demonstrate that late autumn convection in the plume shortly before ice formation significantly reduces friction between the plume and the subjacent sea. The subsequent formation of solid sea ice coverage isolates the plume from wind forcing. These two factors precondition the Ob-Yenisei plume to form an intense buoyancy-driven coastal current below sea ice. As a result, the plume advects eastward to the Laptev Sea through the Vilkitsky Strait during several months in November-February. Eventually, by late winter this huge freshwater volume disappears from the Kara Sea and contributes to freshwater content of the Laptev Sea. The obtained result improves our understanding of freshwater balance of the Kara and Laptev seas, as well as provides an important insight into the large-scale freshwater transport in the Eurasian Arctic, which remain largely unknown during ice-covered season.

## Introduction

River runoff to the Arctic Ocean (4200 km^3^ y^−1^) accounts to 11% of total freshwater discharge to the World Ocean, which provides anomalously large freshwater input per unit area (0.28 m y^−1^) compared to the other oceans (0.08–0.18 m y^−1^)^1–3^. Spreading and transformation of freshwater runoff governs many physical (stratification, vertical mixing, ocean heat flux), chemical (nutrient cycle, acidification), and biological (primary production, food webs) processes in the Arctic Ocean^4–6^. In particular, it maintains salinity-driven density stratification and affects sea ice formation, which is of key importance for the Earth’s albedo and global climate^7–9^.

**Figure 1:**
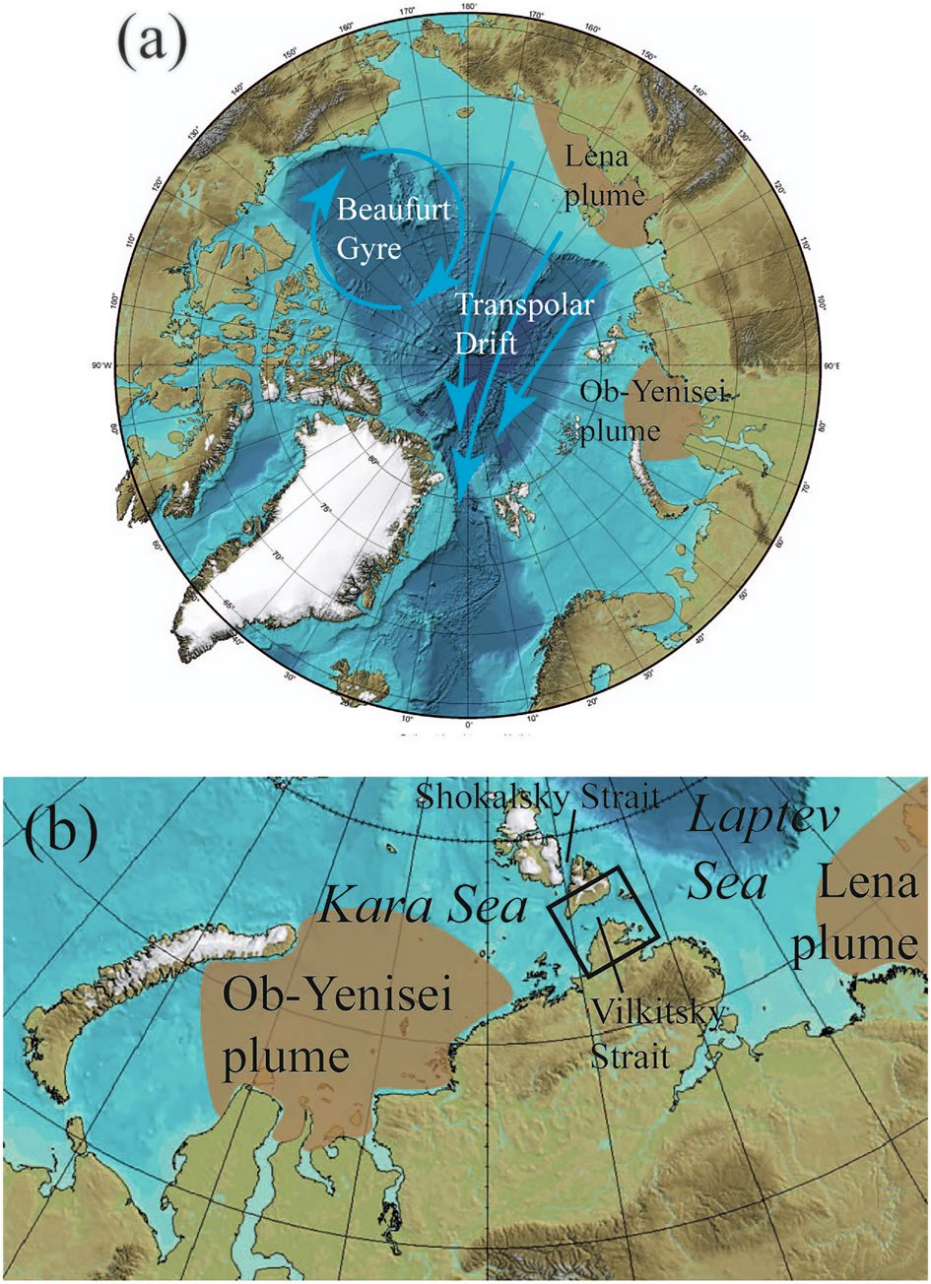
Bathymetry maps of the Arctic Ocean (**a**) and the Kara Sea and the Vilkitsky Strait (b) indicating general surface circulation scheme of the Arctic Ocean (Transpolar Drift and Beaufort Gyre, blue arrows) and average location of the Ob-Yenisei and Lena plumes during ice-free season (brown areas). The black rectangle in panel (**b**) indicates location of the study area in the Vilkitsky Strait shown in Fig. 3a. These maps were generated using IBCAO bathymetry (version 4.2).

The surface circulation in the deep Arctic is dominated by the anticyclonic Beaufort Gyre in the Canadian Basin and the Transpolar Drift, which flows from the northern parts of the East-Siberian and Laptev seas towards the Fram Strait^10^ (Fig. 1a). The surface circulation at the wide Eurasian shelf is determined by spreading of large river plumes, which have significantly different dynamics, as compared to ambient saline sea^11,12^. Large density gradient at the vertical plume-sea interface reduces vertical momentum transport between buoyant plumes and subjacent seawater^13^. As a result, plume dynamics are governed by wind forcing, buoyancy gradients and Coriolis force, while experiencing low influence of ambient sea circulation^14,15^.

The Ob-Yenisei plume (with area up to 250,000 km^2^) located in the Kara Sea and the Lena plume (with area up to 500,000 km^2^) located in the Laptev and East-Siberian seas are the largest plumes in the Arctic Ocean and among the largest in the World Ocean^16–19^ (Fig. 1). These two plumes cover the majority of the Eurasian Arctic shelf during ice-free season in summer and autumn and determine local circulation, which was thoroughly described in previous studies based on extensive in situ measurements^20–25^. Structure and spreading dynamics of these plumes are assumed to be strongly modified in winter and spring due to decrease of river discharge during cold season and isolation of wind influence on river plume by solid ice coverage. However, severe weather and ice conditions in the Kara, Laptev, and East-Siberian seas during cold season are the reasons of dramatic lack of in situ measurements from October to May. As a result, almost nothing is known about the Ob-Yenisei and Lena plumes during ice-covered season that lasts during more than half of the year.

This study is focused on the fate of the Ob-Yenisei plume during ice-covered season. We present the first analysis of large-scale advection of the Ob-Yenisei plume below sea ice based on direct observations of salinity structure in the surface layer in the Kara Sea and the Vilkitsky Strait (Fig. 1b). This study fills a large gap in understanding a key component of large-scale freshwater transport in the Eurasian Arctic Ocean.

## Results

Carmack et al.^26^ argued that discharges of multiple rivers to the Arctic Ocean eventually form buoyancy-driven coastal currents due to their initial deflection to the right by the Coriolis force and the subsequent geostrophic balance of alongshore buoyancy flows. These currents are neither stationary nor continuous, but on the long-term period they are believed to form a large-scale zonal freshwater transport along the Eurasian Arctic and North America Arctic coasts.

Nevertheless, this effect was not ever observed at the Eurasian Arctic coast between two major river plumes, namely, the Ob-Yenisei and Lena plumes. While the Lena plume is advected eastward along the East Siberian coast as a freshened buoyancy current (the Siberian Coastal Current)^27–29^ and northward by the Transpolar Drift after being mixed with saline surface seawater^30^, the long-term fate of the Ob-Yenisei plume still remains unclear. Analogously to the Lena plume, freshwater volume accumulated in the Ob-Yenisei plume could be transported eastward to the Laptev Sea and/or northward to the central part of the Arctic Ocean. However, it is still unclear, which of these two processes dominate.

During ice-free season, the Ob-Yenisei plume has relatively stable area (on the inter-annual time scale) in the central part of the Kara Sea bounded by sharp thermohaline gradient, because thermohaline characteristics of the Ob-Yenisei plume are significantly different from those of the surrounding sea. First, salinities within the plume are < 25–28, while salinities outside the plume are > 32^16^. Second, watershed basins of the Ob and Yenisei rivers are extended several thousands of kilometers southward from the Kara Sea, as a result, temperatures within the Ob-Yenisei plume are greater than in saline seawater during the majority of the ice-free period^31^. Temperatures within the plume decrease from > 10 °C in July to 0.5–1 °C in late autumn. Temperatures below the plume are < 0 °C during the whole year.

The Ob-Yenisei plume could form a narrow (20–30 km wide) freshened buoyancy current, which reaches the Vilkitsky Strait and further inflows to the Laptev Sea with potential to merge with the Lena plume^29,32,33^. This process requires specific wind forcing conditions, namely, strong and long-term southwesterly winds, which occur relatively rarely during ice-free seasons (and could not occur at all at certain years)^29^. Therefore, it results in eastward advection of only small share of the huge freshwater volume accumulated in the Ob-Yenisei plume, while the majority of this volume remains in the central part of the Kara Sea till the end of the ice-free season. Eastward advection of the Ob-Yenisei plume through the Shokalsky Strait located ~ 100–150 km northward from the Vilkitsky Strait (Fig. 1b), as well as northward advection of the Ob-Yenisei plume to the central part of the Arctic Ocean was never observed by in situ measurements.

**Figure 2:**
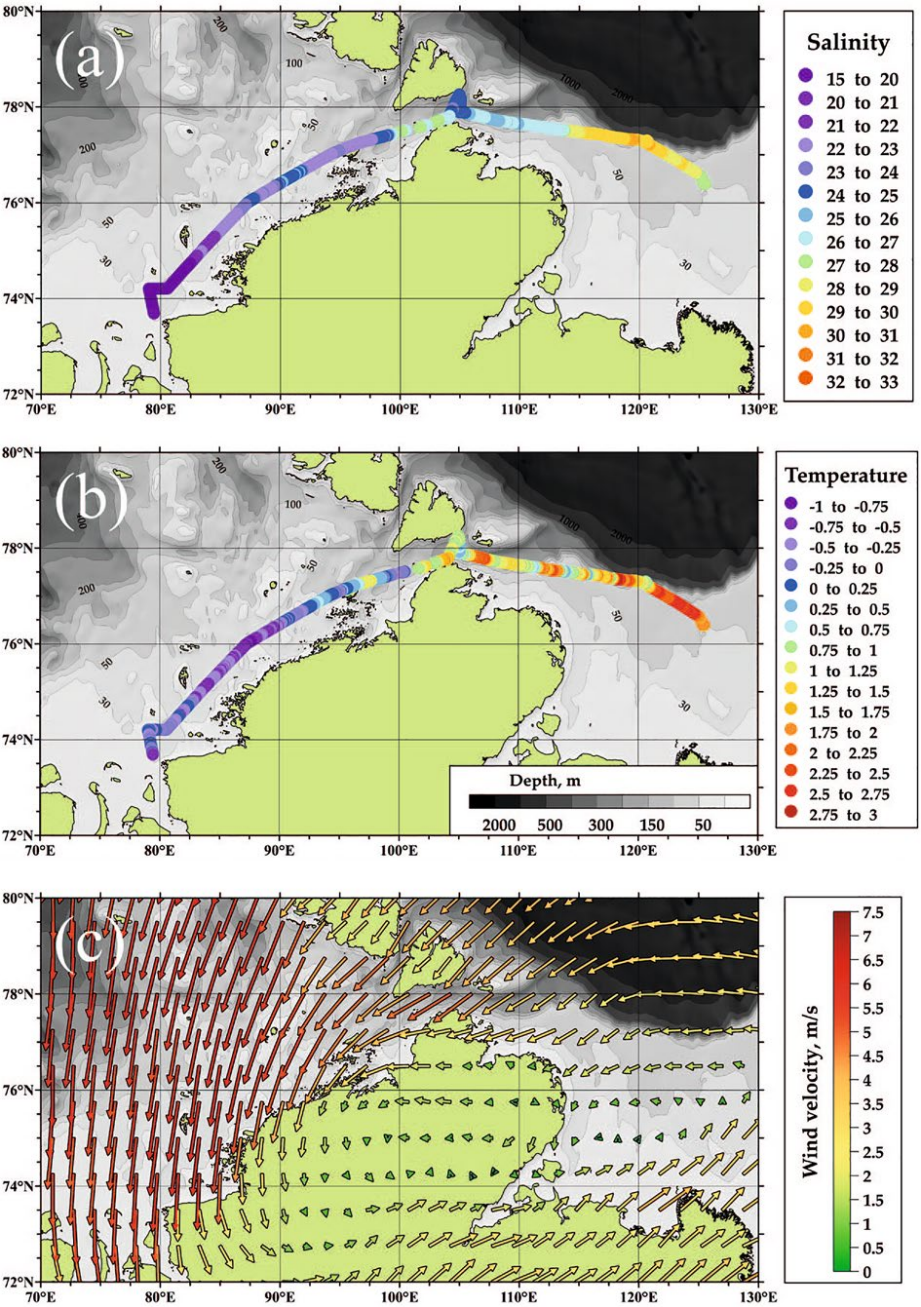
Salinity (**a**) and temperature (**b**) of the surface layer in the Kara and Laptev seas along the ship track on 21–25 October 2020 according to in situ measurements during oceanographic survey. Average wind conditions in the Kara Sea in October 2020 according to ERA5 wind reanalysis (**c**). These maps wer generated using Surfer 8 software (version 25.1).

In situ measurements of thermohaline characteristics of the surface layer performed in the Kara and Laptev seas by a ferry-box system on 21–25 October 2020 revealed low salinities (< 28) (Fig. 2a) and high temperatures (> 0.5 °C) (Fig. 2b) in the central and eastern parts of the Kara Sea, as well as in the northwestern part of the Laptev Sea. These measurements indicate advection of low-saline and warm Ob-Yenisei plume through the Vilkitsky Strait and its further spreading in the Laptev Sea till the longitudes of 110–112°E. However, wind conditions preceding the in situ measurements were not favorable for formation of any alongshore buoyancy current, because northerly and easterly winds dominated in the central and eastern parts of the Kara Sea during the beginning and middle of October 2020 (Fig. 2c). Under typical conditions during ice-free season, these winds tend to keep the Ob-Yenisei plume at the central part of the Kara Sea.

**Figure 3:**
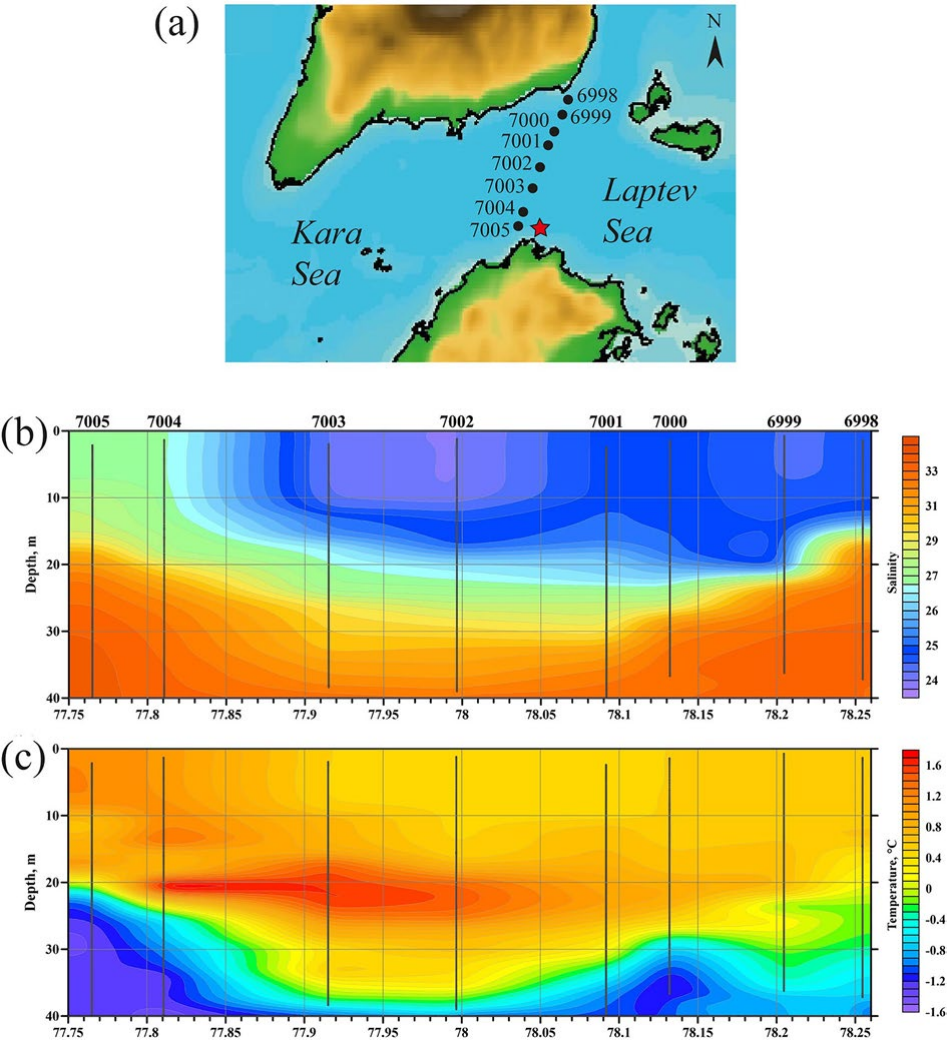
(**a**) Location of the hydrographic stations in October 2020 (black circles) and the mooring station from October 2020 to October 2021 (red star) in the Vilkitsky Strait. Vertical salinity (**b**) and temperature (**c**) structure across the Vilkitsky Strait from surface till the depth of 40 m on 23 October 2020 interpolated from in situ measurements performed at hydrographic stations (indicated by vertical black lines). The map in panel (**a**) was generated using IBCAO bathymetry (version 4.2).

Vertical in situ measurements performed during the oceanographic survey across the Vilkitsky Strait on 23 October 2020 (Fig. 3a) at the very end of the ice-free season (8 days before ice formation) revealed anomalously wide and intense eastward freshwater flow, which was not preconditioned by wind forcing. The whole width of the Vilkitsky Strait was occupied by low-salinity (< 28) (Fig. 3b) and warm (> 0.5 °C) (Fig. 3c) water mass, which expanded from surface to the depth of 15–30 m indicating presence of the Ob-Yenisei plume. The width of the freshened surface layer in the strait observed in October 2020 was ~ 60 km, while during previously reported periods of advection of the Ob-Yenisei plume through the Vilkitsky Strait the width of the freshened flow was ~ 20 km^29^.

The registered presence of distinct eastward flow of the Ob-Yenisei plume to the Laptev Sea under northerly and easterly winds contradicts previous understanding of wind-driven physical background of this process. Below in this paper we solve this question and reveal that under certain conditions eastward spreading of the Ob-Yenisei plume becomes possible without wind forcing. In particular, it becomes possible below sea ice, which is among the key findings of this paper.

**Figure 4:**
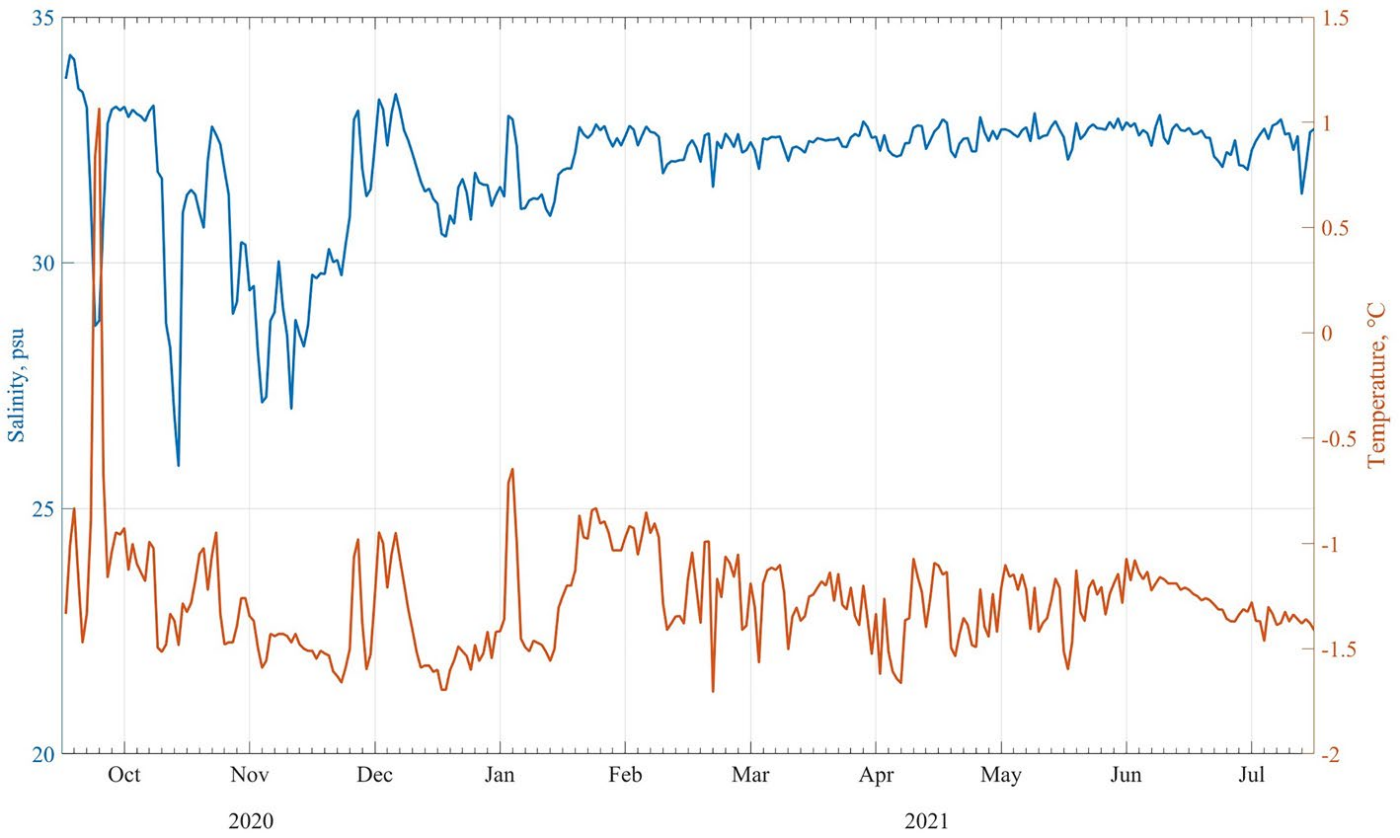
Salinity (blue line) and temperature (orange line) at the depth of 30 m in the Vilkitsky Strait during October 2020–July 2021 according to in situ measurements at the mooring station (indicated by the red star in Fig. 3).

Additionally to in situ measurements, the mooring station was deployed in the southern part of the Vilkitsky Strait (at the distance of 5 km from the Cheluskin Cape) on 5 October 2020 (red star in Fig. 3a). It provided information about variability of salinity and temperature during 10 months (October 2020–July 2021) including the full ice-covered season (Fig. 4). These measurements revealed several periods of abrupt salinity drops (till 26–31 at the depth of 30 m), which occurred from late October till the middle of January. In total these periods lasted during ~ 45 days indicating frequent advection of the Ob-Yenisei plume through the Vilkitsky Strait.

**Figure 5:**
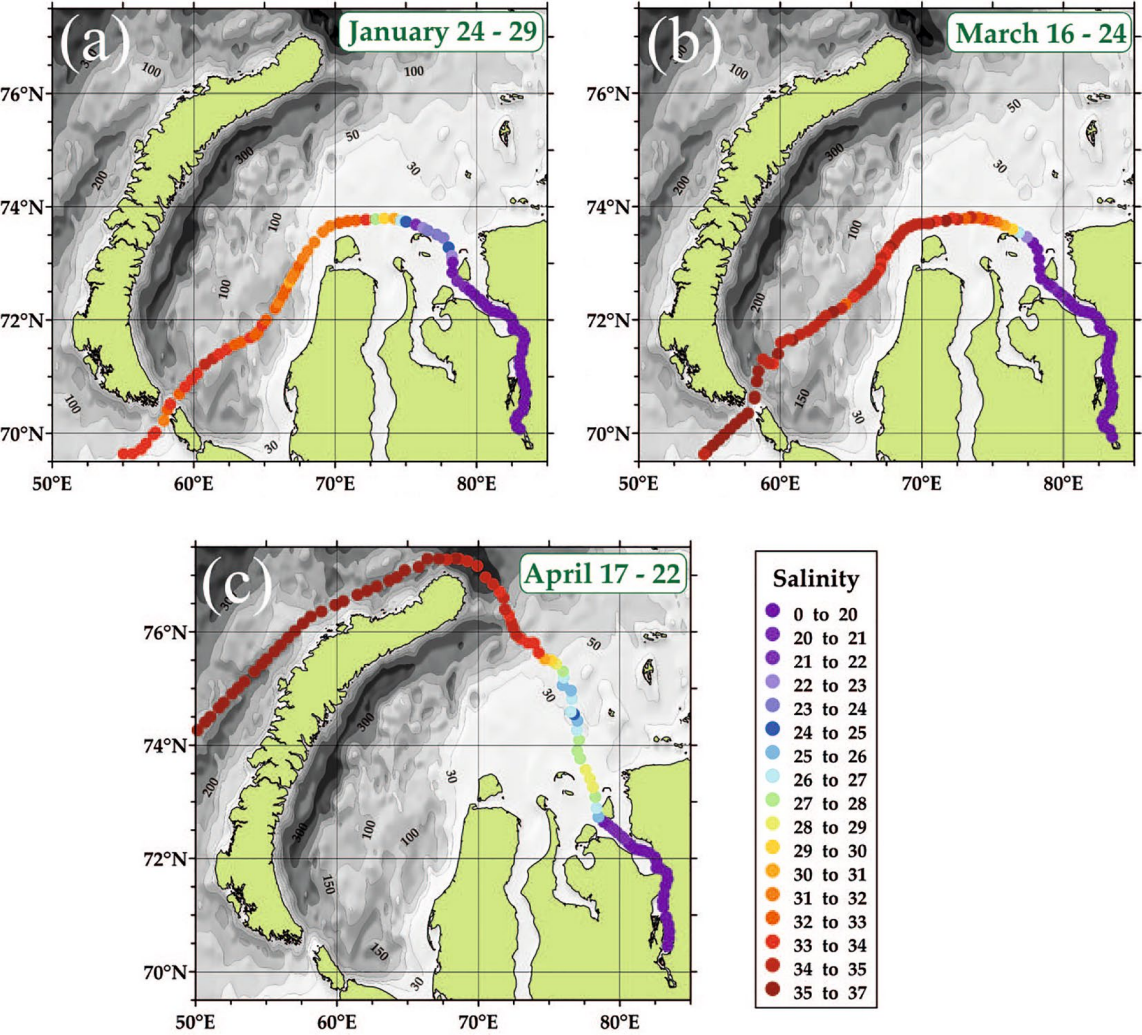
Salinity in the surface layer in the central part of the Kara Sea during ice-covered season according to in situ measurements by ferry-box systems on 24–29 January 2021 (**a**), 16–24 March 2021 (**b**), 17–22 April 2021 (**c**). These maps were generated using Surfer 8 software (version 25.1).

Measurements at the mooring station in the Vilkitsky Strait during ice-covered season were supported by ferry-box measurements of salinity in the surface layer in the Kara Sea, which were performed in January–April 2021 (Fig. 5). These measurements revealed absence of the Ob-Yenisei plume in the central part of the sea manifested by high salinities (> 28). Low salinities were observed only within the Yenisei Gulf indicating advection of huge freshwater volume contained in the Ob-Yenisei plume off the Kara Sea.

## Discussion

Direct measurements shortly before and during the cold season in the Kara and Laptev seas and the Vilkitsky Strait revealed the following features of salinity distribution in the surface layer: (1) propagation of freshened water from the Kara Sea to the Laptev Sea through the Vilkitsky Strait shortly before ice formation, (2) low salinities in the Vilkitsky Strait during October–January followed by high salinities during February–July, (3) high salinities in the central part of the Kara Sea from late January till late April. These results are evidences of eastward advection of the Ob-Yenisei plume through the Vilkitsky Strait during ice-covered season. The Ob-Yenisei plume was leaking from the Kara Sea to the Laptev Sea from early October till the middle of January.

We presume that in late January this processes finished because the whole plume was transferred either eastward to the Laptev Sea, or northward to the central part of the Arctic Ocean (however, the latter case was not covered yet by our in situ measurements and remains beyond the current study). Nevertheless, we lack in situ measurements during winter and spring in the northeastern part of the Kara Sea, in particular, at the area between the Gulf of Ob and the Vilkitsky Strait. As a result, some freshwater volume from the Ob-Yenisei plume could possibly remain in winter and spring in the northeastern part of the Kara Sea. However, there are indirect evidences that the whole plume leaks through the Vilkitsky Strait to the Laptev Sea. First, in situ measurements in August^16^, which are the earliest available measurements in this area, reveal absence of low salinities in the northeastern part of the Kara Sea. Second, regular accumulation of freshened surface layer in the northeastern part of the Kara Sea would strongly affect seafloor sediments in this area, which was not observed by previous studies^34,35^. Nevertheless, this question could be properly solved only by in situ measurements, which is a part of our planned future work.

**Figure 6:**
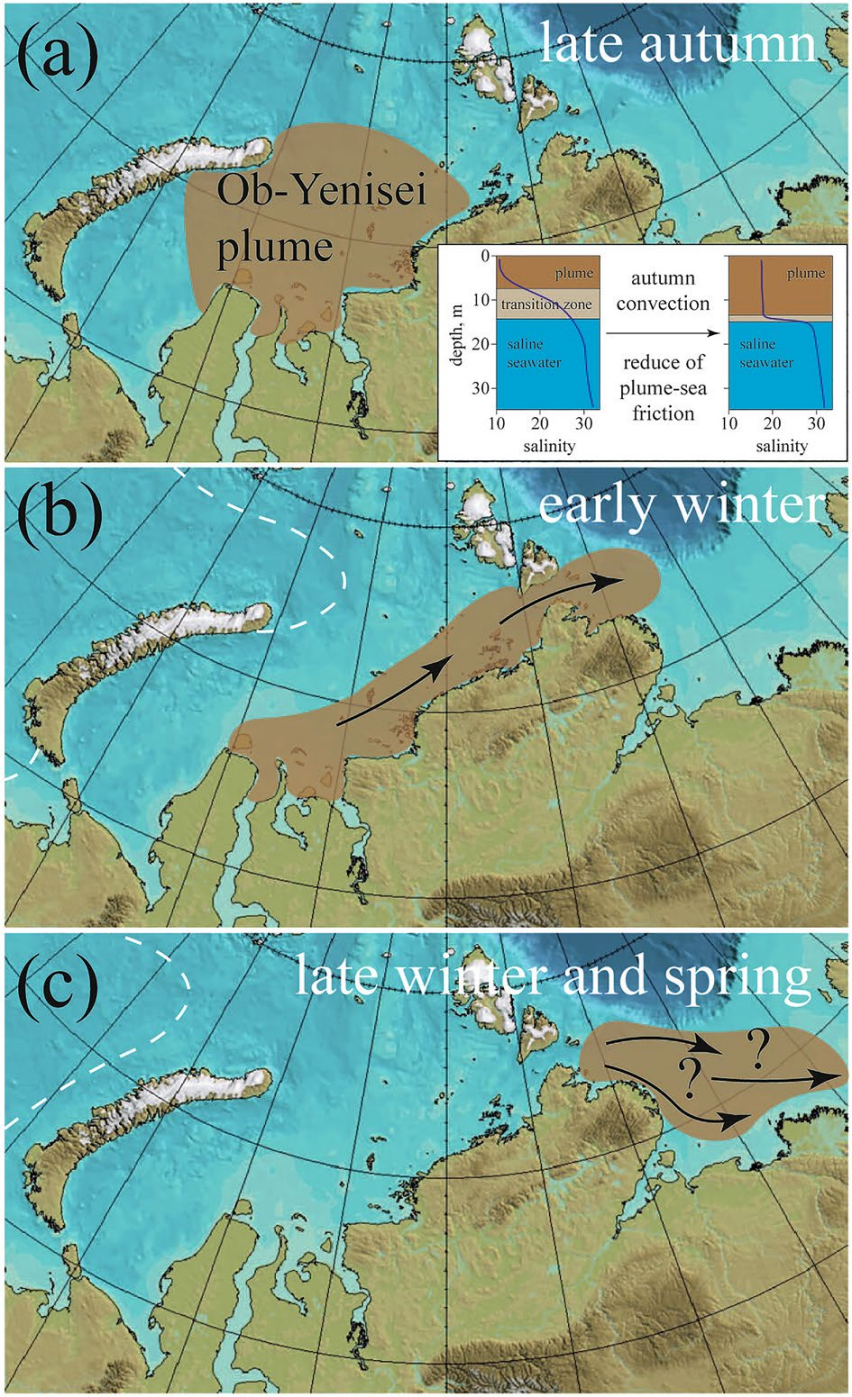
Scheme of formation of intense zonal freshwater transport through the Vilkitsly Strait during ice-covered season: (**a**) vertical convection within the Ob-Yenisei plume in late autumn shortly before ice formation, (**b**) advection of the Ob-Yenisei plume from the Kara Sea to the Laptev Sea as a coastal buoyancy-driven current during early winter, (**c**) absence of the Ob-Yenisei plume in the Kara Sea in late winter and spring. The inset in panel (**a**) demonstrates changes in the vertical salinity structure in the central part of the Kara Sea as a result of autumn convection. White dashed lines in panels (**b**), (**c**) indicate average locations of the seasonal sea ice edge. Note that location and advection of the Ob-Yenisei plume shown in panel (**c**) is a hypothesis, because this area is still not covered by in situ measurements during ice-covered season. These maps were generated using IBCAO bathymetry (version 4.2).

Osadchiev et al.^31^ recently described abrupt transformation of vertical structure of the Ob-Yenisei plume shortly before ice-covered season caused by autumn convection. Atmospheric cooling of the freshened surface layer in the Kara Sea in late autumn results in, first, vertical homogenization of the Ob-Yenisei plume and, second, formation of a very sharp vertical salinity gradient at the plume-sea interface. It significantly affects dynamics of the Ob-Yenisei plume once it becomes isolated from wind forcing after the beginning of the ice-covered season. Formation of a very sharp vertical salinity gradient at the bottom boundary of the Ob-Yenisei plume dramatically reduces friction between the plume and the subjacent sea. Before convection starts, salinity at the plume-sea interface increases from 20 to 30 at vertical distance of ~ 5–10 m, while convection reduces this distance to 1–2 m (inset in Fig. 6a). Vertical eddy viscosity, which determines friction strength between two sea layers, could be estimated using the equation A_v_ = 10^–4^ + 9·10^–4^/(1 + 0.3·Ri)^1/2^, where Ri is the Richardson number equal to the relation between buoyancy frequency and vertical velocity shear^36^. Autumn convection increases vertical density gradient, and, therefore, the Richardson number changes from 1.1 to 8 indicating transition of the plume dynamics to the buoyancy-driven alongshore flow. Vertical eddy viscosity reduces from 8.8·10^–4^ to 5.9·10^–4^, i.e., friction at the plume-sea interface decreases by 1.5 times.

Due to the lack of direct velocity measurements in the Vilkitsky Strait, we use a model of two-layer buoyancy-driven coastal current to estimate the alongshore flow velocity of the Ob-Yenisei plume once it becomes isolated from wind forcing^37^. Conservation of potential vorticity of the geostrophic flow is described by the equation $$\frac{{{\text{f}} - \frac{{\partial {\text{u}}}}{{\partial {\text{y}}}}}}{{\text{h}}} = \frac{{\text{f}}}{{\text{H}}}$$, where h is the plume layer thickness, u is the alongshore velocity, f is the Coriolis parameter, H is the plume depth. Geostrophic balance of the stable flow yields $${\text{u}} = - \frac{{g^{\prime } }}{{\text{f}}}\frac{{\partial {\text{h}}}}{{\partial {\text{y}}}}$$, hence $$\frac{{\partial^{2} {\text{h}}}}{{\partial {\text{y}}^{2} }} - \frac{{\text{h}}}{{{\uplambda }^{2} }} = - \frac{{\text{H}}}{{{\uplambda }^{2} }}$$, where $${\uplambda } = \sqrt {{\text{g}}^{\prime } {\text{H}}} /{\text{f}}$$ is the baroclinic Rossby radius of deformation between the plume with the density of $${\uprho }_{1}$$ and the ambient sea with the density of $${\uprho }_{0}$$, $${\text{g}}^{\prime } = {\text{g}}\left( {{\uprho }_{0} - {\uprho }_{1} } \right)/{\uprho }_{0}$$ is the reduced gravity between the plume and the ambient sea. The general solution of the latter equation is the following $$  {\text{h}}\left( {\text{y}} \right) = {\text{Ee}}^{{{\raise0.7ex\hbox{${\text{y}}$} \!\mathord{\left/ {\vphantom {{\text{y}} \lambda }}\right.\kern-\nulldelimiterspace} \!\lower0.7ex\hbox{$\lambda $}}}}  + {\text{Fe}}^{{{\raise0.7ex\hbox{${ - {\text{y}}}$} \!\mathord{\left/ {\vphantom {{ - {\text{y}}} \lambda }}\right.\kern-\nulldelimiterspace} \!\lower0.7ex\hbox{$\lambda $}}}}  + {\text{K}} $$, where E, F, K are the constants of integration, hence $$ {\text{u}}\left( {\text{y}} \right) =  - \frac{{{\text{g}}^{\prime } }}{{\text{f}}}\left( {\frac{{\text{E}}}{\lambda }{\text{e}}^{{{\raise0.7ex\hbox{${\text{y}}$} \!\mathord{\left/ {\vphantom {{\text{y}} \lambda }}\right.\kern-\nulldelimiterspace} \!\lower0.7ex\hbox{$\lambda $}}}}  - \frac{{\text{F}}}{\lambda }{\text{e}}^{{{\raise0.7ex\hbox{${ - {\text{y}}}$} \!\mathord{\left/ {\vphantom {{ - {\text{y}}} \lambda }}\right.\kern-\nulldelimiterspace} \!\lower0.7ex\hbox{$\lambda $}}}} } \right) $$. We apply the following boundary conditions: u(0) = 0, h(0) = H, h(L) = 0, velocity u is continuous across y = L, where L is the steady flow plume width. Our in situ data show that the Ob-Yenisei plume depth is $${\text{H}} = 30{\text{ m}}$$, plume width at the Vilkitshy Strait is $${\text{L}} = 65{\text{ km}}$$, mean densities of the plume and the subjacent seawater below sea ice taken from in situ data yield the mean plume flow velocity equal to $${\text{U }} = { }0.48{\text{ m}}/{\text{s}}$$.

The coastal current model used in this study is based on^38^. The main advantage of this model is that it is simple and can provide estimations of average velocity without usage of complex and computationally expensive numerical models. However, a disadvantage is that the model is one-dimensional and only depends on cross-shore dimension, i.e., distance from the shoreline, so it cannot account for vertical effects like drag from sea ice. The coastal current model could be used to estimate the average coastal current velocity based on measured density gradients and depth of the plume. However, the effect of drag from the sea ice cover on the flow needs to be added. We are studying a coastal current during the ice season, so we reproduce the influence of landfast ice cover. We parameterize ice cover to act as a solid motionless plate^39^. Below ice is a two-layered flow, with fresher water over denser water. We assume steady laminar flow in the upper layer, with no mixing between layers. The interface between the layers is assumed to be at rest^40^. Laminar flow between plates has a parabolic velocity profile ${\text{u}}\left( {\text{z}} \right) = \frac{1}{{2{\upmu }}}\frac{{\partial {\text{p}}}}{{\partial {\text{y}}}}\left( {{\text{Hz}} - {\text{z}}^{2} } \right),$ where µ is the viscosity, $\frac{{\partial {\text{p}}}}{{\partial {\text{y}}}}$ is the pressure gradient, H is the depth of the freshened upper layer. This type of the flow has a maximum velocity at the intermediate depth. The average velocity can be related to the maximum velocity of the flow in the following way ${\text{U}}_{{\text{c}}} = \frac{2}{3}{\text{U}} = 0.32\;{\text{m}}/{\text{s}}$, where ${\text{U}} = 0.48{\text{ m}}/{\text{s}}$ is the average velocity of the costal current model. Applying no-slip conditions at both the sea ice and the interface between two layers, we obtain a minimum estimate of the average current velocity.

The Kara and Laptev seas are connected by two straits, namely, 50–80 km wide Vilkitsky Strait in the south and 15–40 km wide Shokalsky Strait in the north (Fig. 1b). In this study, we focus on measurements in the Vilkitsky Strait, which evidence advection of the Ob-Yenisei plume through this strait during ice-covered season. However, similar process could also occur in the Shokalsky Strait located ~ 100–150 km northward from the Vilkitsky Strait. Recently, Makhotin et al.^41^ and Savelieva et al.^42^ described multiple in situ measurements performed in the northeastern part of the Shokalsky Strait (the Baranov Cape at the Bolshevik Island of the Severnaya Zemlya Archipelago). They report that salinity in the surface layer exceeded 30–31 during January–May in 2014–2016 and 2019. This result reveals that advection of the Ob-Yenisei plume from the Kara Sea to the Laptev Sea occurs only through the Vilkitsky Strait, this flow does not passes through the Shokalsky Strait. This result is indirect evidence that northward advection of the Ob-Yenisei plume to the central part of the Arctic Ocean does not occur at least in the northeastern part of the Kara Sea, otherwise it could be registered in the Shokalsky Strait. However, this question should be addressed primarily on the base of specific in situ measurement in the northern part of the Kara Sea performed during the whole ice season. To the extent of our knowledge, these measurements were not yet performed, but are within the scope of our future work.

Once we estimate average velocity of eastward alongshore flow of the Ob-Yenisei plume below sea ice as 0.32 m/s, the whole plume (with area of 200,000–250,000 km^2^ in autumn^16^) will pass through the Vilkitsky Strait (with average width of 65 km) during 110–140 days. This estimation is in a good agreement with the in situ measurements at the mooring station in the Vilkitsky Strait, which demonstrated presence of the plume in the straight during 3–4 months from the middle of October till January–February.

Figure 6 summarizes the fate of the Ob-Yenisei plume during ice-covered season. Atmospheric cooling and sea convection in the Kara Sea in late autumn shortly before ice formation increase lateral plume-sea pressure gradient and reduce vertical plume-sea friction (Fig. 6a). As a result, convection is a key process that preconditions large-scale freshwater transport of river plumes in the Eastern Arctic below sea ice. After formation of stable ice coverage and isolation from wind forcing, the Ob-Yenisei plume forms an eastward alongshore buoyant current and leaks to the Laptev Sea through the Vilkitsky Strait during early winter (Fig. 6b). By late winter the Ob-Yenisei plume disappears from the Kara Sea and is absent there till the beginning of freshet period at the Ob and Yenisei rivers (Fig. 6c).The fate of the Ob-Yenisei plume in the Laptev Sea after it flowed through the Vilkitsky Strait remains unknown due to absence of in situ measurements in this area during ice-covered season. If we assume that the Ob-Yenisei plume will continue its eastward alongshore propagation below sea ice, it could reach the central part of the Laptev Sea (Fig. 6c). Then it will merge with the Lena plume before the beginning of ice melting and start of the freshet period at the Lena River in June-July. In this case, the huge freshwater volume accumulated during ice-free season in the Kara Sea in the next summer and autumn will contribute to the freshened surface layer in the Laptev Sea.

Previous in situ measurements in the Eastern Arctic demonstrated that freshwater volume accumulated during ice-free season in the Kara Sea (~ 1000 km^3^) is similar to total river runoff to this sea during summer-autumn freshet period. On the opposite, freshwater volume accumulated during ice-free season in the Laptev and East-Siberian seas (1500–2000 km^3^) is much greater than total annual river runoff to these seas (~ 1000 km^3^)^17,43,44^. This difference could be explained by the advection of the Ob-Yenisei plume from the Kara Sea to the Laptev Sea during winter and spring.

River runoff to the Arctic Ocean increased by 5–10% during the last century and is predicted to continue increasing in future as a result of the ongoing climate change^45,46^. The majority of this runoff increase occurs during winter and spring^47^ and influences regional processes in the Kara and Laptev seas including plankton communities^48–52^, carbonate system^53,54^, and marine pollution^55,56^. This fact highlights the importance of study of large-scale freshwater cycle in the Arctic during ice-covered season, in particular, possible eastward alongshore advection of the Lena plume below sea ice, which remains unknown.

## Data and methods

Hydrographic in situ data used in this study were collected during an oceanographic survey in the Vilkitsky Strait and adjacent areas of the Kara and Laptev seas onboard the research vessel “Akademik Mstislav Keldysh” on 21–25 October 2020. The vertical thermohaline structure from surface to sea bottom was measured using CTD instruments (SBE 911plus) at 0.2-m spatial resolution. Salinity measurements in the surface layer (at the depth of 4 m) were performed by a ferry-box system equipped by thermosalinograph (SBE21) every 15 s.

Temperature and salinity measurements in the Vilkitsky Strait at the depth of 30 m during ice-covered season were performed at the mooring station equipped by a CTD logger (centi-CTD StarOddi). The mooring station was installed at the distance of 5 km from the Cheluskin Cape at the most narrow part of the Vilkitsky Strait and performed measurements from October 2020 to July 2021. The measurements were performed every 6 h.

Salinity measurements in the surface layer (at the depth of 4 m) in the Kara Sea during ice-covered season were performed by a ferry-box system equipped by salinity logger (centi-CT StarOddi). The ferry box was installed on the ice-breaking container vessel “Monchegorsk”, which crossed the Kara Sea from the Kara Gates Strait to the Yenisei Gulf on 24–29 January and 16–24 March 2021 and from the Zhelaniya Cape to the Yenisei Gulf on 17–22 April 2021. The measurements were performed every 1 h.

Wind forcing conditions were examined using ERA5 atmospheric reanalysis with a 0.25° spatial and hourly temporal resolution^57^.

## Data Availability

The datasets analysed during the current study are available from the corresponding author on reasonable request.
